# High proviral load of human T cell lymphotropic virus type-1 facilitates coronary artery diseases

**DOI:** 10.22038/ijbms.2020.36317.8649

**Published:** 2020-04

**Authors:** Farnaz Mozayeni, Seyed Abdolrahim Rezaee, Farahzad Jabbari Azad, Mahmoud Shabestari, Reza Faridhosseini, Houshang Rafatpanah, Hadis Yousefzadeh, Yousef Ali Garivani, Lida Jarahi, Narges Valizadeh, Faezah Sabet, Sharare Moshirahmadi, Fatemeh Sadat Mohammadi, Mohammad Shabestari

**Affiliations:** 1 Allergy Research Center, School of Medicine, Mashhad University of Medical Sciences, Mashhad, Iran; 2 Immunology Research Center, Inflammation and Inflammatory Disease Division, Mashhad University of Medical Sciences, Mashhad, Iran; 3 Department of Cardiology, Emam Reza Hospital, School of Medicine, Mashhad University of Medical Sciences, Mashhad, Iran; 4 Immunology Research Center, Bu Ali Research Institute, Student Research Committee, School of Medicine, Mashhad University of Medical Sciences, Mashhad, Iran; 5 Addiction Research Center, Mashhad University of Medical Sciences, Faculty of Medicine, Mashhad, Iran

**Keywords:** Cardiac involvement, Coronary artery disease, HTLV-1, HTLV-1 Proviral load, Real-time PCR

## Abstract

**Objective(s):**

Coronary artery disease (CAD) is known as a life threatening disease, worldwide. In this study the role of HTLV-1 infection was evaluated on cardiac involvement in an endemic region of northeastern Iran.

**Materials and Methods:**

Serologic and molecular tests for HTLV-1 infection were carried out in subjects who had coronary angiography. A real-time PCR, TaqMan method, to quantify HTLV-1 proviral load (PVL), and routine hematological and biochemical tests were performed for study subjects.

**Results:**

Twenty nine patients were HTLV-1+CAD+ and 13 cases were HTLV-1+CAD-. Although, there were no significant differences for risk factors like FBS, HDL, triglyceride, systolic and diastolic blood pressure (Cbp, Dbp), waist circumference (WC), hip circumference (WL), cholesterol (*P*=0.003), and LDL (*P*=0.007) levels, and monocyte count (*P*=0.05) had meaningful differences. The mean HTLV-1 PVL in HTLV-1+CAD+ subjects was 992.62±120 which was higher compared with HTLV-1+CAD- group (406.54±302 copies/104 PBMCs). Moreover, HTLV-1 PVL in males (833±108) was lower compared with females (1218±141 copies/104 PBMCs) (*P*=0.05). Patients with HTLV-1-PVL of more than 500 copies/104 had more diffused atherosclerosis plaque than patients with less than 500 (OR=6.87, 95% CI=1.34-35.05; *P*=0.016). Furthermore, patients with diffused coronary atherosclerosis had significantly higher levels of HTLV-1 PVL than patients with middle, proximal, and normal location of coronary sclerotic lesions (*P*<0.05).

**Conclusion:**

Taken together, in endemic area, HTLV-1 infection, more likely is a facilitating factor for heart complications and the high HTLV-1 PVL might affect CAD manifestations.

## Introduction

Cardiovascular diseases in developing countries are the most common cause of death (1). According to the World Health Organization (WHO) reports, about 17.3 million people died from cardiovascular diseases in 2008, worldwide (2). Coronary artery disease (CAD) is the most prevalent form of cardiovascular diseases. CAD is characterized by accumulation of plaques, made up of cholesterol deposits within the walls of the coronary arteries, resulting in the narrowing or blockage of arteries, and eventually leading to angina and myocardial infarction (3, 4). This inflammatory disease is accompanied by immunologic mechanisms and metabolic risk factors (5, 6). Traditional risk factors of CAD are dyslipidemia, diabetes mellitus, hypertension, smoking, metabolic syndrome, obesity, and drug addiction (3). 

The associations of some infections have been recently confirmed in the pathogenesis of atherosclerotic plaques. Furthermore, inflammation was involved as one of the definite probable causes in this process (7). On the other hand, a great deal of evidence has demonstrated a central role of inflammation due to a thin layer of white streaks on the artery wall which caused the development of a complex series of cellular events, including T-lymphocytes, astrocytes, high macrophage foam cell count (histological markers for plaque vulnerability), basophiles, and platelet interactions with vascular surfaces besides fatty streaks, and subsequently led to formation of athermanous plaques (6, 8-10). 

Previous studies have investigated the role of inflammatory mediators, cytokines, and some vasoactive substances as important risk factors of atherosclerosis, including TNF-α, IL-1, IL-6, IFN-γ, and C-reactive protein (CRP). High blood levels of these cytokines have been associated with atherosclerosis, autoimmune disease, chronic inflammatory diseases, and cancers (11-13). 

 Patients suffering from acute myocardial infarction exhibit high concentrations of pro-inflammatory cytokines like TNF-α and IL-6 (14). Larger IFN-γ pool in blood of acute coronary syndrome patients were demonstrated before (15). Finally, synergy between CRP and inflammatory mediators including IFN-γ and lipopolysaccharide (LPS) might play a direct pro-thrombotic role in the pathogenesis of coronary atherosclerosis and its acute complications by increasing monocyte/macrophage of monocyte tissue factor (16).

The possible roles of infections inducing atherosclerosis were suggested more than 200 years ago by several authors. The main organisms that were recently suggested to involve in CAD manifestation include cytomegalovirus (CMV), herpes simplex virus (HSV-1), hepatitis virus, and bacteria like* Chlamydia pneumonia*, *Porphromonus gingivalis *and* Helicobacter pylori *(17). 

Human T-cell lymphotropic virus type 1 (HTLV-1) is a retrovirus that allows the majority of HTLV-I-infected individuals remain healthy carriers, whereas less than 5% of the infected individuals develop acute T Cell leukemia/lymphoma (ATLL) or HTLV-1-associated myelopathy/tropical spastic paraparesis (HAM/TSP) (18-23). Northeast of Iran (Khorasan provinces), were reported as an endemic areas with a prevalence of 2.3%-3.4% (19-24). Cardiac involvement in HTLV-1-infected individuals has been reported in some studies (10, 25). Our previous serologic and molecular (PCR) study on HTLV-1 infection in subjects with heart complications, showed prevalence of 13.5% and 10.5%, respectively, which is nearly three times more than the prevalence of this infection in the general population of this city (3.4%). The probable correlation between HTLV-1 infection and coronary vessel involvement could be introduced as an associated risk factor for coronary artery disease with the mechanism of vascular inflammation (26). According to our previous studies (10, 25) and considering the Razavi and North Khorasan provinces, with more than 7 million population (National Population and Housing Census 2011), as an endemic area for HTLV-1, in this study HTLV-1 proviral load as a major determinant of HTLV-I infection in patients with CAD along with some other biochemical and hematological factors were investigated. To have a close look, the possible effect of HTLV-1 infection on CAD, the type of coronary involvements, the location of sclerotic lesions, and the diffusion of atherosclerotic plaques were also considered.

## Materials and Methods

***Ethics and study population***

The Research Ethics Committee of Mashhad University of Medical Sciences approved this study (IR.MUMS.REC.900177) and a written informed consent form was obtained from each participant. Among 73 HTLV-1 infected patients who referred for coronary artery angiography, 42 subjects obtained the appropriate criteria to include in this study. Then, HTLV-1 proviral load was measured and coronary artery involvements were determined based on the angiography reports. According to the HTLV-1 proviral load, patients were divided into two groups: more than 500 HTLV-1 proviral copies/10^4 ^PBMCS group and less than 500 group. Healthy carrier was attributed to patients with positive HTLV-1 patients and negative CAD. 

***Data collection procedures***

A blood sample (10 ml) was collected from each subject for evaluation of risk factors in cardiovascular disease. Biochemical parameters including low density lipid (LDL), high density lipid (HDL), cholesterol (Ch), triglycerides, and fasting blood sugar (FBS) levels as common cardiovascular disease risk factors, as well as cell blood count (CBC), systolic and diastolic blood pressure (Cbp, Dbp), waist circumference (WC), and hip circumference (WL) were measured. A cardiologist examined the patients and an echocardiogram was performed to determine the coronary lesions and the last angiography film was inspected by two specialists separately to distinguish involvement in coronary arteries. All participants completed a check list including demographic information and medical history. 

***Serological and molecular assays***

Firstly, the HTLV-1 infection was identified by the ELISA method (Dia.Pro, Italy) and then confirmed by the PCR technique. Peripheral blood mononuclear cells (PBMCs) were isolated by standard Ficoll–Hypaque (Sigma, USA) density centrifugation. Cells were washed twice by Phosphate buffer slain (PBS) (Sigma, USA) and PBMCs were kept at –20 ^°^C until further analysis. Genomic DNA was extracted on the same day from PBMCs using DNeasy Kit (GeneBio, Korea) according to the manufacturer’s instructions. To confirm HTLV-1 infection, conventional PCR was carried out for *Tax* and the long terminal repeat (LTR) using the following primers: *Tax* forward primer (5’- AGGGTTTGGACAGAGTCTT-3’), Tax reverse primer (5’-AAGGACCTTGAGGGTCTTA), LTR forward primer (5’-ATAAGCTCAGACCTCCGGG-3’), and LTR reverse primer (5’-GGATGGCGGCCTCAGGTAGG-3’). The PCR products were analyzed on 1.5% agarose gel, stained with ethidium bromide, and then visualized under UV light (27).

***HTLV-1 proviral load measurement***

Quantitative real-time PCR (qRT-PCR) was carried out using a commercial absolute quantification kit (Novin Gene, Iran) by Rotor Gene 6000 software (Qiagen, Germany). The test was performed with the Universal Master Mix (Takara, Japan) to measure HTLV-1 proviral load in PBMCs with the Taqman method. The HTLV-1 proviral copies number was reported as an actual amount of cellular DNA by means of the quantification of the albumin gene as the reference gene. Then *Tax *DNA of HTLV-1 and human albumin DNA concentrations were calculated from two five-point standard curves. The normalized value of the HTLV-1 proviral load was calculated as the ratio of (HTLV-1 DNA copies number / albumin DNA copies number / 2) × 10^4^ and expressed as the number of HTLV-1 provirus per 10^4^ PBMCs (25). 

***Statistical analysis***

Data were statistically analyzed using SPSS software version 16.0 (SPSS, Chicago, USA). Descriptive data were summarized as mean±standard deviation (SD), standard error of mean (SEM), and percent. Normality of the data was checked prior to data analysis by the Kolmogorov-Smirnov test. Independent samples *t*-test was used to compare HTLV-1 proviral load between HTLV-1^+^CAD^+^ and HTLV-1^-^CAD^-^) patients. One-way ANOVA test was performed to analyze the association between CAD and type of coronary vessel involved with HTLV-1 proviral load in patients with CAD. 

The independent samples *t*-test was applied for analyzing the association between diffusion of atherosclerotic involvement and HTLV-1 proviral load in the HTLV-1^+^CAD^+^ group. Chi-square or Fisher’s exact test was used for determining association between confounding variables and coronary artery disease. The odd ratios (ORs) and their 95% confidence interval (95% CI) were estimated. The results were considered statistically significant if *P*≤0.05. 

## Results

***Patients’ characteristics***

**Figure 1.**
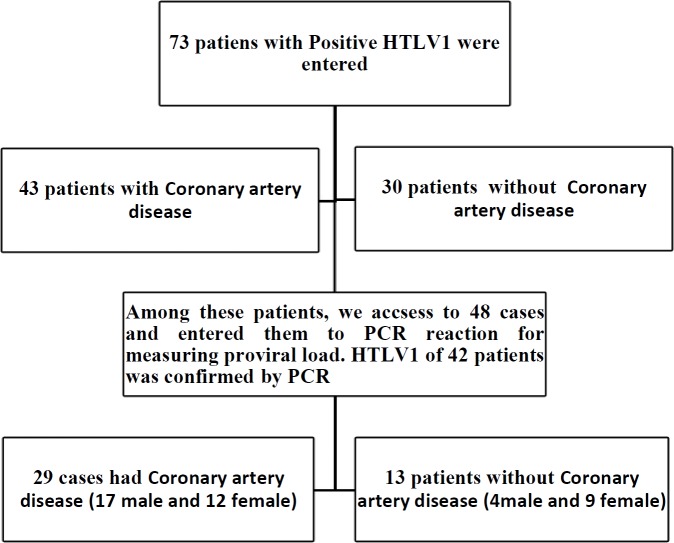
The study flowchart of sampling

Among 48 patients who participate in this study, 29 patients were HTLV-1^+^CAD^+^ and 13 cases were HTLV-1^+^CAD^-^ (Figure 1). There was no significant difference in mean age of CAD^+^ (65±8 years) and CAD^-^ (62±10 years) patients. Table 1 shows the demographic characteristics of the CAD^+^ and CAD^-^ groups. This table represents that there are not any significant differences in demographic variables between the two studied groups. However, mild pulmonary illness was detected in CAD^+^ group not in CAD^-^. Furthermore, significant differences in risk factors for cardiovascular diseases such as FBS, HDL, WC, WL, Cbp, Dbp, and just (Ch) (*P*=0.003) and LDL (*P*=0.007) were meaningful. Comparing the mean of CBC results indicates that there was only significant difference for monocytes (*P*=0.05) between CAD^+^ and CAD^-^ groups. Clinical and laboratory findings of HTLV-1^+^CAD^+^ and HTLV-1^+^CAD^-^ are summarized in Table 2. Of note, that common CAD confounding variables did not affect the findings of the study and the two groups were matched for statistical analysis. 

***Coronary artery involvement***

The number and types of involved coronary arteries, the location of sclerotic lesions, and the diffusion of atherosclerotic involvement were determined (Table 3). According to the angiography reports, the involvement of coronary arteries for one vessel, two vessels, and three vessels were seven (24.13%), seven (24.13%), and 15 (51.72%) of the patients, respectively. Types of involved coronary arteries were as follows: involvement of left circumflex artery (LCX) in three patients (10.3%), left anterior descending coronary artery (LAD) in three patients (10.3%), and right coronary artery (RCA) in one patient (3.4%). Furthermore, the involvements in LAD-LCX, LAD-RCA, LCX-RCA, and LAD-LCX-RCA were present in two (6.9%), four (13.8%), two (6.9%), and 14 (48.3%) patients, respectively. 

***Localization of sclerotic lesions***

Coronary artery involvement was investigated at the beginning, end, and middle of the RCA, LCX, and LAD (Table 3). The involvement in more than five regions of the RCA, LCX, and LAD was considered as diffuse coronary atherosclerosis. The findings showed a statistically significant difference between the location of LCX and RCA sclerotic lesions (*P*=0.02) and LAD and RCA lesions (*P*=0.001). Generally, 14 patients (48.3%) had diffused coronary atherosclerosis; however, non-diffuse coronary artery disease was observed in 15 (51.7%) of patients.

***HTLV-1 proviral load in heart complications***

The mean HTLV-1 proviral load in HTLV-1^+^CAD^+^ and HTLV-1^+^CAD^-^ were 992.62±12 and 406.54±602 copies/10^4 ^PBMCs, respectively, in which the difference was statistically different (*P*=0.045). 

The proviral load of HTLV-1^+^CAD^+^ subjects in males (833±108copies/10^4 ^PBMCs) was significantly lower than females (1218±141) (*P*=0.05). While in HTLV-1^+^CAD^-^ patients, the males (760±812 copies/10^4 ^PBMCs) had significantly higher proviral load compared to females (249±453) (*P*=0.034). 

Furthermore, the statistically significant correlation between the mean of HTLV-1 proviral load and coronary artery involvement was (*P*=0.027, R=0.03). The patients with three-vessel involvement had a mean HTLV-1 proviral load of 937.77±189.26 copies/10^4 ^PBMCs, which is significantly higher than those with one vessel involvement (767.23±300.71) or two vessels involved (409.98±174.93). Besides, the findings showed that statistically significant correlations were observed between HTLV-1 proviral load of coronary artery involvement of the RCA vessel, in the beginning of the RCA vessel (*P*=0.002), and diffuse coronary atherosclerosis status (*P*=0.031). 

**Figure 2:**
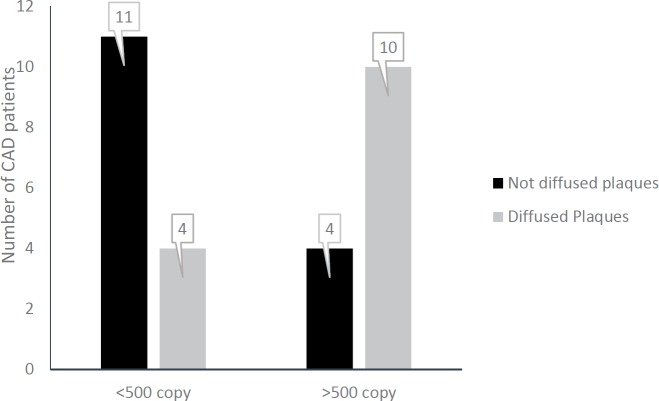
The amount of HTLV-1 proviral loads and atherosclerosis diffusion. Proviral load of more than 500 strongly affects atherosclerosis diffusion. PVL presented as copies /104 PBMCs

**Figure 3:**
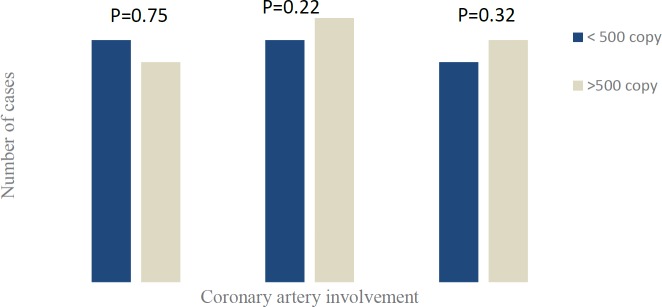
Association between coronary artery involvement and HTLV-1 proviral load. LCX (left circumflex artery), LAD (left anterior descending coronary artery), and RCA (right coronary artery). Proviral load presented as HTLV-1 proviral copies /104 PBMCs

HTLV-1 infected patients were put in two groups according to the proviral load , patients with less than 500 copies/10^4 ^PBMCs (L group) and equal or more than 500 copies (M group). Coronary artery disease patients who had a proviral load of more than 500 copies/10^4 ^PBMCs had higher estimated risk of diffuse coronary artery disease than patients with less than 500 copies (*P=*0.016, OR= 6.87, 95% CI=1.34-35.05, Figure 2). Therefore, patients with proviral load of more than 500 (proviral virus/10^4^ PBMCs) were 6.87 times more in risk of diffused atherosclerosis plaque formation than patients with less than 500 copies or HTLV-1 healthy carrier. HTLV-1 proviral load association with coronary artery involvement is depicted in Figure 3. However, high proviral load (M group) was attributed to LAD, RCA, and LCX vessels, and low proviral load (L group) to LAD, LCX and RCA.

**Figure 4:**
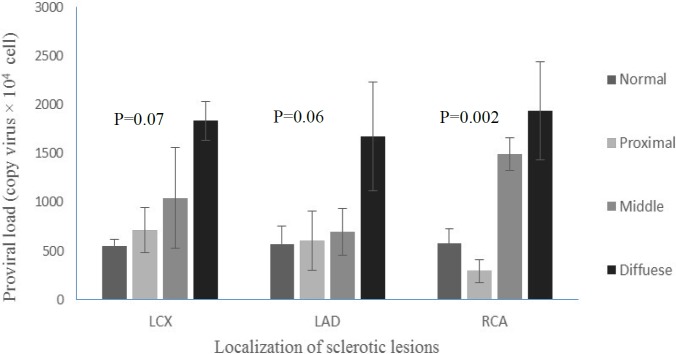
The effect of HTLV-1 proviral load on localization of the sclerotic lesions in patients with different involvements of coronary vessels: LCX (left circumflex artery), LAD (left anterior descending coronary artery), and RCA (right coronary artery)

Strikingly, statistical analysis findings demonstrated that there are significant differences in HTLV-1 proviral load in patients with different localization of sclerotic lesions in involvement vessels (Figure 4). Figure 4 shows that patients with diffused location of coronary atherosclerosis (LCX, LAD, and RCA) had significantly higher levels of HTLV-1 proviral load than patients with middle, proximal, and normal location of coronary sclerotic lesions, respectively. 

## Discussion

The present study demonstrated that there is an association between high viral loads of HTLV-1 infection and diffusion of atherosclerosis plaque in coronary artery disease. Few studies are available on effect of HTLV-1 infection and cardiac involvement, of which most are case reports. In 1993 Gabarre *et al.* reported a 60-year-old woman with cardiac failure due to aortic and mitral regurgitations from our study region (Mashhad, Iran). Assessing her samples by PCR test showed that she was positive for HTLV-1. It was the first report of an isolated lymphomatous cardiac valve involvement without the other cardiac abnormalities (28). There is not any relevant study to address the promoted molecular pathway to cardiovascular disease positivity with high HTLV-1 proviral load. 

In other retroviral diseases, for example in HIV infection, the role of ongoing viral transcribes in vascular dysfunction and cardiovascular disease are reported (29). This may be partially explained by the presence of HIV-infected reservoir cells by HIV early-gene encoded proteins (Tat and Nef) as reservoir cells and associated cytokine signaling are important in the development and promoting of cardiomyopathy. 

In HTLV-1 infection, considering the role of proviral load and clinical value, Sabouri *et al.* (30) found that HTLV-1 provirus load in Iranian HAM/TSP patients is one of the major risk factors for developing HAM/TSP. Besides research (29) showed that infected cells in HIV patients hide in tissues such as the lymphatic system, and its low-level transcriptions continues even after years of antiretroviral therapy but their relevance for disease is unclear. 

In the present study, the proviral load of HTLV-1 was assessed in HTLV-1 positive patients with normal or CAD^+^ complications. It can be suggested that a high proviral load of HTLV-I had a strong association with diffusion of atherosclerosis plaque in CAD^+^ patients (OR=6.87). Furthermore, the findings showed that there were significant differences in HTLV-1 proviral load among various sclerotic plaque localizations. This was due to higher levels of HTLV-1 proviral load in diffused localization of plaque in LCX, LAD, and RCA vessel than patients with middle, proximal, and normal coronary sclerotic involvement lesions, respectively (*P*<0.05).

Previous study by FaridHosseini *et al.* (25) reported that sero-prevalence of HTLV-I in subjects with heart complications in Neyshabour, Iran was nearly three times more than the general population of this city (10.5 % vs 3.4%). On the other hand in a study in Brazilian HAM/TSP patients suggested a better cardiovascular risk profile than healthy subjects (31). Firstly, this study only evaluated the cardiovascular risk profile in a rehabilitation center in HAM/TSP patients. Secondly, the coincidences of two different associated HTLV-1 diseases are rare. Thirdly, the methodology of the study is totally different compared to the present study. However, there are no direct studies on cardiac involvement and HTLV-1 infection, furthermore, an uncommon autopsy case of ATLL with massive cardiac involvement (32), a HTLV-1 associated lymphoma with expanded calcification in the heart (33), and a HTLV-1 infection with multiple organ failure and symptoms of advanced cardiac insufficiency were reported (34). These reports documented that the cardiac involvement along with HTLV-1 infection associated to adult ATLL. The cardiac autopsy pulmonary in the patients with ATLL had suggested increased risk of cardiac involvement (32).

Therefore, it seems that proviral load has a direct role in HTLV-1 associated diseases. Our previous studies explained that host epigenetic changes and virulence factors of HTLV-1 such as proviral load, *Tax,* and *HBZ* are the main factors for associated disease manifestations. Among them, HTLV-1 proviral load, *Tax,* and *HBZ* expression could be used as prognostic factors or monitoring markers for the efficiency of therapeutic regimes (26, 35-37). 

Collectively, many studies suggested that the endothelial cells may be infected with HTLV-1 and disrupt the integrity of the vessels, particularly in blood brain barrier (BBB), towards HAM/TSP manifestations. The recent studies had more emphasis on endothelial cells and HTLV-1 infected cells interactions. For example, the changes in CCR1, CCR2, CXCR5, CXCR6, CXCL9, and CXCL10 showed the tendency of HTLV-1 infected cells to the vessels, particularly, BBB (38). Another study showed that the HTLV-1-Tax virulence factor increases activated leukocyte cell adhesion molecule (ALCAM/CD166) expression, which facilitates the recruitment of infected cells across the BBB endothelium (39).

 Taken together, these findings showed that HTLV-1 infected T-cells have a very close interaction with endothelial cells and by inducing inflammatory reactions in the surface of the particular vessels might facilitate CAD. Therefore, evaluating the impact of HTLV-1-TCD4-IFN-γ^+^ or unique infected T cell subset of CCR4^+^ CD4^+^ CD25^+^ with CAD are suggested. Moreover, repeated cross-sectional studies are recommended to understand the dynamics of HTLV-1 epidemic in high prevalence areas. Finding such risk factors, for example viruses, should allow us suitable intervention strategies for prevention or therapy. Of importance, due to the previous documentation on association of HTLV-1 genotype and risk of HAM/TSP, the authors suggest further studies could concentrate on correlation between the incidence of CAD or its severity and HTLV-1 genotype or with other associated diseases.

**Table 1:** Demographic characteristics of the HTLV-1+CAD+ and HTLV-1+CAD- groups

Variables	**CAD+ (N=29)**	**CAD- (N=13)**	***P*** **-value**
Dyslipidemia	9 (31)	5 (38.5)	0.31
Hypertension	11 (37.9)	6 (46.2)	0.61
History of heart disease in the family	6 (20.7)	1 (7.7)	0.61
History of high cholesterol	6 (20.7)	3 (23.1)	0.86
Diabetes	5 (17.2)	2 (15.4)	0.88
Kidney disease	4 (13.8)	2 (15.4)	0.89
Smoking	2 (6.9)	1 (7.7)	0.14
Autoimmune disease	8 (27.6)	4 (30.8)	0.55
Respiratory disease	7 (24.1)	0	0.052
Thyroid disease	1 (3.4)	0	0.49
Hormonal disease	1 (3.4)	1 (7.7)	0.49

Number in parenthesis shows percentages of involved patients in each column

CAD: Coronary artery disease

**Table 2:** Clinical and laboratory findings of the HTLV-1+CAD+ and HTLV-1+CAD- groups

**Variable**	**CAD+ (Mean±SE) **	**CAD- (Mean±SE) **	***P*** **-value**
**FBS**	114.34±11	102.54±13	0.198
**Cholesterol**	182.07±36	218.54±28	0.003
**TG**	143.93±58	132.15±56	0.543
**HDL**	48.62±8	50.69±11	0.525
**LDL**	104.28±25	127.15±21	0.007
**WC**	95.69±13	97.15±11	0.737
**WL**	101.97±9	101.92±6	0.988
**Cbp**	148.97±18	144.44±21	0.541
**Dbp**	84.14±6	78.89±8.4	0.294
Neutrophils	53.66±9	56.46±8	0.361
Lymphocytes	41.17±9	36.46±8	0.133
Monocytes	2.86±1	4.69±2	0.050
Eosinophils	2.31±1	2.54±1	0.617
WBC	6.27±1	6.16±1	0.810
**RBC**	4.74±0.5	4.71±0.3	0.846
**Hb**	13.75±1	13.52±1	0.611
**HCT**	41.79±4	40.59±3	0.347
**MCH**	29.08±1	28.64±1	0.484
**MCV**	88.26±5	86.6±3	0.187
**MCHC**	33.9±1	32.23±1	0.428
Platelets	229.66±5	22.45±15	0.681

FBS: Fasting blood sugar; TG: Triglyceride; LDL: Low density lipid; HDL: High density lipid; WC: Waist circumference; WL: Hip circumference; Cbp: Systolic blood pressure; Dbp: Diastolic blood pressure; WBC: White blood cell; RBC: Red blood cell; Hb: Hemoglobin; HCT: Hematocrit; MCV: Average Red blood cell size; MCH: Hemoglobin amount per red blood cell; MCHC: Hemoglobin concentration per red blood cell; CAD: Coronary artery disease

**Table 3:** Coronary artery involvement and its localization among study participants

**Variables**				**Number (%)**	
Coronary involvement	Female		One-vessel Two-vessel Three-vessel	1(8.3) 4 (33.3) 7 (58.3)
	Male		One-vessel Two-vessel Three-vessel	6 (36.3) 3 (17.6) 8 (47.1)
Localization of sclerotic lesions				
Left circumflex Artery		Beginning of vessel Middle of vessel Diffuse vessel Normal vessel		10 (34.5) 5 (17.2) 6 (20.7) 8 (27.6)
Left anterior descending coronary artery		Beginning of vessel Middle of vessel Diffuse vessel Normal vessel		8 (27.6) 6 (20.7) 9 (31.7) 6 (20.7)
Right coronary artery		Beginning of vessel Middle of vessel Diffuse vessel Normal vessel		10 (34.5) 3 (10.3) 8 (27.6) 8 (27.6)

## Conclusion

It is more likely that infecting particular endothelial cells with HTLV-1 or cell-cell interactions between endothelial cells and HTLV-1 infected TCD4+ disrupt endothelium integrity, which can induce inflammatory reactions toward inflammatory diseases such as HAM/TSP (40) or CAD manifestations. Therefore, targeting these interactions could be a new path for preventing or reducing the development of such HTLV-1 associated diseases. In conclusion, as some authors suggested, HTLV-1 associated diseases may be due to clinical or subclinical inflammatory reactions in the vessels due to HTLV-1 infected TCD4 interactions with endothelial cells.

## References

[1] Nizal Sarrafzadegan M, Sadeghi M, Shahram Oveisgharan M, Marshall T (2013). Incidence of cardiovascular diseases in an Iranian population: the Isfahan Cohort Study. Arch Iran Med.

[2] World Health Organization (2011). Global status report on noncommunicable diseases 2010 World Health Organization.

[3] Ebrahimi M, Kazemi-Bajestani S, Ghayour-Mobarhan M, Ferns G (2011). Coronary artery disease and its risk factors status in Iran: A review. Iran Red Crescent Med J.

[4] Alizadehsani R, Habibi J, Bahadorian B, Mashayekhi H, Ghandeharioun A, Boghrati R (2012). diagnosis of coronary arteries stenosis using data mining. J Med Signals Sens.

[5] Silventoinen K, Magnusson PK, Tynelius P, Batty GD, Rasmussen F (2009). Association of body size and muscle strength with incidence of coronary heart disease and cerebrovascular diseases: a population-based cohort study of one million Swedish men. Int J Epidemiol.

[6] Libby P (2006). Inflammation and cardiovascular disease mechanisms. Am J Clin Nutr.

[7] Miller GE, Freedland KE, Duntley S, Carney RM (2005). Relation of depressive symptoms to C-reactive protein and pathogen burden (cytomegalovirus, herpes simplex virus, Epstein-Barr virus) in patients with earlier acute coronary syndromes. Am J Cardiol.

[8] Siddiqui T, Dikshit D (2013). Platelets and atherothrombosis: causes, targets and treatments for thrombosis. Curr Med Chem.

[9] Liao X, Sluimer JC, Wang Y, Subramanian M, Brown K, Pattison JS (2012). Macrophage autophagy plays a protective role in advanced atherosclerosis. Cell Metab.

[10] Shabestari MM, Jabbari F, Gohari B, Moazen N, Azizi H, Moghiman T (2011). Coronary artery angiographic changes in veterans poisoned by mustard gas. Cardiology.

[11] Siegel D, Devaraj S, Mitra A, Raychaudhuri SP, Raychaudhuri SK, Jialal I (2013). Inflammation, atherosclerosis, and psoriasis. Clin Rev Allergy Immunol.

[12] Klingenberg R, Hansson GK (2009). Treating inflammation in atherosclerotic cardiovascular disease: emerging therapies. Eur Heart J.

[13] Myers GL, Rifai N, Tracy RP, Roberts WL, Alexander RW, Biasucci LM (2004). CDC/AHA Workshop on markers of inflammation and cardiovascular disease application to clinical and public health practice: Report from the laboratory science discussion group. Circulation.

[14] Hassanzadeh M, Faridhosseini R, Mahini M, Faridhosseini F, Ranjbar A (2006). Serum levels of TNF-, IL-6, and selenium in patients with acute and chronic coronary artery disease. Iran J Immunol.

[15] Bergstrom I, Backteman K, Lundberg A, Ernerudh J, Jonasson L (2012). Persistent accumulation of interferon-gamma-producing CD8+CD56+ T cells in blood from patients with coronary artery disease. Atherosclerosis.

[16] Nakagomi A, Freedman SB, Geczy CL (2000). Interferon-gamma and lipopolysaccharide potentiate monocyte tissue factor induction by C-reactive protein: relationship with age, sex, and hormone replacement treatment. Circulation.

[17] Shah PK (2001). Link between infection and atherosclerosis: who are the culprits: viruses, bacteria, both, or neither?. Circulation.

[18] Rafatpanah H, Farid R, Golanbar G, Azad FJ (2006). HTLV-I Infection: virus structure, immune response to the virus and genetic association studies in HTLV-I-infected individuals. Iran J Allergy Asthma Immunol.

[19] Treviño A, Aguilera A, Caballero E, Benito R, Parra P, Eiros JM (2012). Trends in the prevalence and distribution of HTLV-1 and HTLV-2 infections in Spain. Virol J.

[20] Vakili R, Sabet F, Aahmadi S, Boostani R, Rafatpanah H, Shamsian A (2013). Human T-lymphotropic Virus Type I (HTLV-I) Proviral Load and Clinical Features in Iranian HAM/TSP Patients: Comparison of HTLV-I Proviral Load in HAM/TSP Patients. Iran J Basic Med Sci.

[21] Azarpazhooh MR, Hasanpour K, Ghanbari M, Rezaee SR, Mashkani B, Hedayati-Moghaddam MR (2012). Human T-lymphotropic virus type 1 prevalence in Northeastern Iran, Sabzevar: an epidemiologic-based study and phylogenetic analysis. AIDS Res Hum Retroviruses.

[22] Rafatpanah H, Hedayati-Moghaddam MR, Fathimoghadam F, Bidkhori HR, Shamsian SK, Ahmadi S (2011). High prevalence of HTLV-I infection in Mashhad, Northeast Iran: a population-based seroepidemiology survey. J Clin Virol.

[23] Hedayati-Moghaddam M, Fathimoghadam F, Mashhadi IE, Soghandi L, Bidkhori H (2011). Epidemiology of HTLV-1 in Neyshabour, Northeast of Iran. Iran Red Crescent Med J.

[24] Foroughipour M, Azad FJ (2013). Outcome of intravenous immunoglobulin-transmitted HTLV-I, hepatitis B, hepatitis C, and HIV infections. Iran J Basic Med Sci.

[25] FaridHosseini R, Jabbari F, Shabestari M, Rezaee SA, Gharivani Y, Valizadeh N (2013). Human T lymphotropic virus type I (HTLV-I) is a risk factor for coronary artery disease. Iran J Basic Med Sci.

[26] Rafatpanah H, Rezaee A, Etemadi MM, Hosseini RF, Khorram B, Afsahr L (2012). The impact of interferon-alpha treatment on clinical and immunovirological aspects of HTLV-1-associated myelopathy in northeast of Iran. J Neuroimmunol.

[27] Rafatpanah H, Torkamani M, Valizadeh N, Vakili R, Meshkani B, Khademi H (2016). Prevalence and phylogenetic analysis of HTLV-1 in a segregated population in Iran. J Med Virol.

[28] Gabarre J, Gessain A, Raphael M, Merle-Beral H, Dubourg O, Fourcade C (1993). Adult T-cell leukemia/lymphoma revealed by a surgically cured cardiac valve lymphomatous involvement in an Iranian woman: clinical, immunopathological and viromolecular studies. Leukemia.

[29] Wang T, Yi R, Green LA, Chelvanambi S, Seimetz M, Clauss M (2015). Increased cardiovascular disease risk in the HIV-positive population on ART: potential role of HIV-Nef and Tat. Cardiovasc Pathol.

[30] Sabouri AH, Saito M, Usuku K, Bajestan SN, Mahmoudi M, Forughipour M (2005). Differences in viral and host genetic risk factors for development of human T-cell lymphotropic virus type 1 (HTLV-1)-associated myelopathy/tropical spastic paraparesis between Iranian and Japanese HTLV-1-infected individuals. J Gen Virol.

[31] Prado F, Prado R, Ladeia AMT (2017). Cardiovascular risk profile in patients with myelopathy associated with HTLV-1. Braz J Infect Dis.

[32] Iemura A, Yano H, Kojiro M, Nouno R, Kouno K (1991). Massive cardiac involvement of adult T-cell leukemia/lymphoma An autopsy case. Arch Pathol Lab Med.

[33] Daisley H, Charles WP (1993). Fatal metastatic calcification in a patient with HTLV-1-associated lymphoma. West Indian Med J.

[34] Daisley H, Charles W (1997). Cardiac involvement with lymphoma/leukemia: a report of three autopsy cases. Leukemia.

[35] Boostani R, Vakili R, Hosseiny SS, Shoeibi A, Fazeli B, Etemadi MM (2015). Triple therapy with prednisolone, pegylated interferon and sodium valproate improves clinical outcome and reduces human T-cell leukemia virus type 1 (HTLV-1) proviral load, tax and HBZ mRNA expression in patients with HTLV-1-associated myelopathy/tropical spastic paraparesis. Neurotherapeutics.

[36] Rafatpanah H, Rezaee A, Etemadi MM, Hosseini RF, Khorram B, Afsahr L (2012). The impact of interferon-alpha treatment on clinical and immunovirological aspects of HTLV-1-associated myelopathy in northeast of Iran. J Neuroimmunol.

[37] Akbarin MM, Rahimi H, Hassannia T, Shoja Razavi G, Sabet F, Shirdel A (2013). Comparison of HTLV-I proviral load in adult T cell leukemia/lymphoma (ATL), HTLV-I-associated myelopathy (HAM-TSP) and healthy carriers. Iran J Basic Med Sci.

[38] Rafatpanah H, Felegari M, Azarpazhooh MR, Vakili R, Rajaei T, Hampson I (2017). Altered expression of CXCR3 and CCR6 and their ligands in HTLV-1 carriers and HAM/TSP patients. J Med Virol.

[39] Curis C, Percher F, Jeannin P, Montange T, Chevalier SA, Seilhean D (2016). Human T-lymphotropic virus type 1-induced overexpression of activated leukocyte cell adhesion molecule (ALCAM) facilitates trafficking of infected lymphocytes through the blood-brain barrier. J Virol.

[40] Afonso PV, Ozden S, Cumont MC, Seilhean D, Cartier L, Rezaie P (2008). Alteration of blood-brain barrier integrity by retroviral infection. PLoS Pathog.

